# Management of dysmenorrhea through yoga: A narrative review

**DOI:** 10.3389/fpain.2023.1107669

**Published:** 2023-03-30

**Authors:** Divya Kanchibhotla, Saumya Subramanian, Deeksha Singh

**Affiliations:** Research Department, Sri Sri Institute for Advanced Research, Bangalore, India

**Keywords:** asanas, dysmenorrhea, meditation, menstrual pain, pranayama, yoga, yoga nidra

## Abstract

Menstrual pain also known as dysmenorrhea is one of the most common and underrated gynecological disorders affecting menstruating women. Although the symptoms and impact might vary greatly, it is defined by cramps in the lower abdomen and pain that radiates to lower back and thighs. In some cases it is also accompanied by nausea, loose stool, dizziness etc. A primary narrative review was conducted on the impact of yoga on dysmenorrhea experienced by women. The English-language literature published until 2022 was searched across databases such as PubMed, Google Scholar and Scopus. “Yoga” and “menstrual pain” OR “dysmenorrhea” were used as keywords for the search across several databases. A total of 816 title searches were obtained across all the database searches. This review article included 10 studies based on the selection criteria. The studies examined the impact of a varied set of 39 *asanas*, 5 *pranayamas* and *Yoga nidra* on dysmenorrhea. The studies demonstrated a significant relief in dysmenorrhea among those who practiced Yoga (*asanas/pranayama/yoga nidra*) with improved pain tolerance and reduced stress levels. Regulating the stress pathways through yoga was found to be a key in regulating hormonal balance and reduction in dysmenorrhea.

## 1. Introduction

Dysmenorrhea is a common issue in menstruating women where the most typical symptoms are cramps and lower abdominal pain. Several experts agree that an aberrant increase in the endometrium's synthesis and vasoactive prostaglandins may cause myometrial hyperactivity, uterine tissue ischemia and discomfort, causing the monthly pain (1). Dysmenorrhea is also accompanied by a number of other premenstrual disorders. Primary dysmenorrhea is a common disorder affecting the Quality of Life (QOL) of many women due to intense pain and related social and psychological implications (2). Appropriate therapy and management are required due to the decreased quality of life, ineffective working hours, and mood swings caused by dysmenorrhea.

In order to improve women's health, several complementary and alternative strategies have been employed to reduce dysmenorrhea, which includes the use of medicinal herbs with analgesic properties (3). Typically, non-steroid anti-inflammatory medications or oral contraceptives are used to treat dysmenorrhea (4). Transcutaneous electrical nerve stimulation, acupuncture, acupressure, topical heat, behavioral interventions, relaxation, herbal and dietary therapy are just a few of the alternatives for dysmenorrhea that are receiving more attention in recent years (5). Among the several complementary therapies accepted, practice of yoga has been added to the list. Yoga is a type of mind-body exercise that combines physical body with a conscious mental emphasis. Yoga is regarded as one of the best holistic stress management approaches since it causes a series of physiological changes in the body that lessen the stress and pain response (6). In case of dysmenorrhea, yoga suppresses the pain by lowering the level of prostaglandin production and myometrial ischemia (1, 2).

## 2. Physiology of the pain

**Figure 1:**
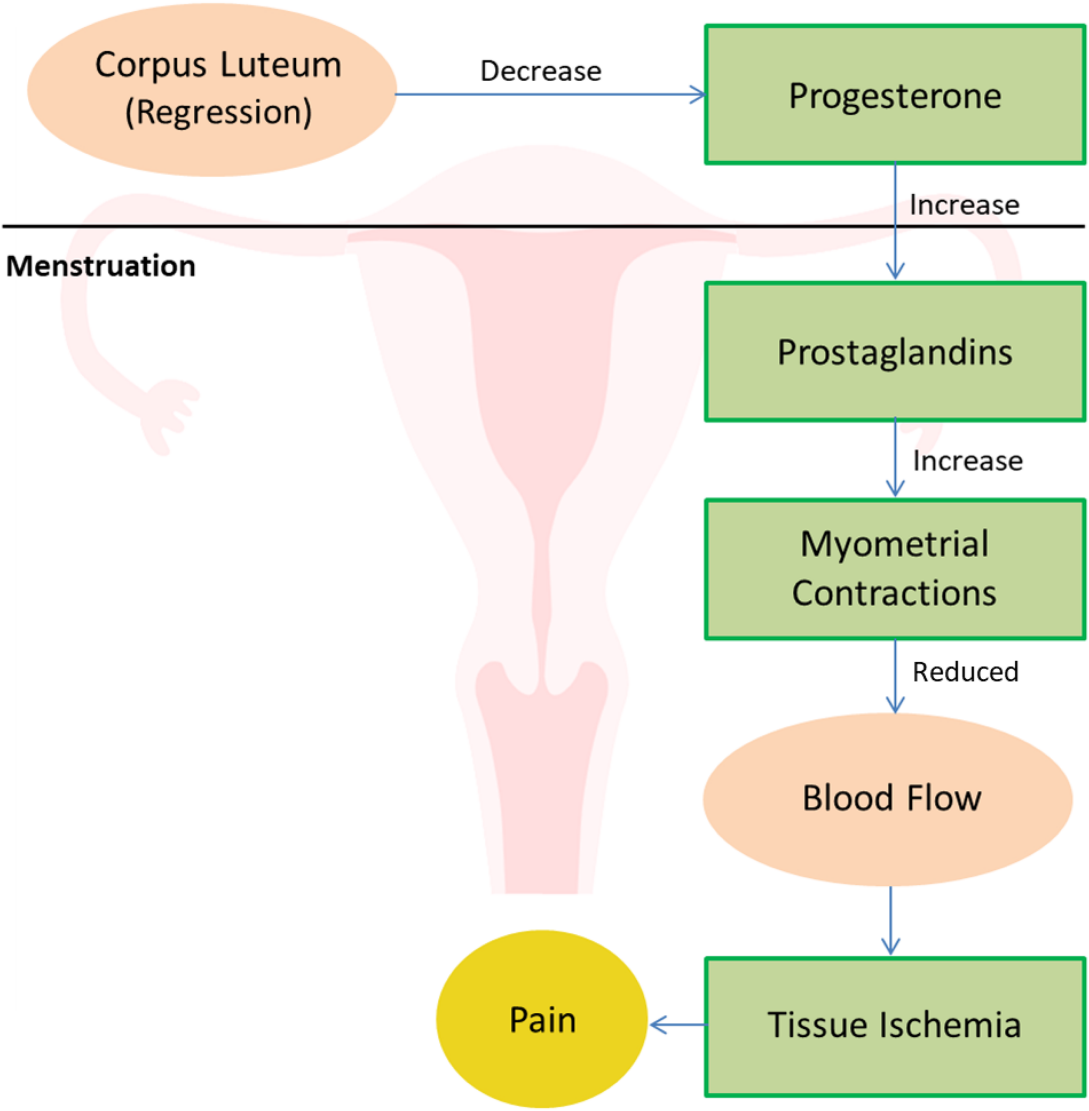
Physiology of dysmenorrhea.

Over the years, a number of hypotheses have been put forth to evaluate the cause of primary dysmenorrhea. Experimental and clinical research has revealed the release of uterine prostaglandins which play a significant role in the progression of primary dysmenorrhea (7). NSAIDs nonsteroidal anti-inflammatory drugs have shown to be effective in reducing the prostaglandin levels so produced during the bleeding (8). As a result of endometrial shedding during menstruation, prostaglandins are released along with the blood. This is accompanied by other enzyme release that breaks down the cell membranes (9). This release of prostaglandin stimulates myometrial contraction and constricts small endometrial blood vessels, resulting in tissue ischemia, endometrial disintegration, bleeding and pain (10) (Figure 1). Another study also suggested an upregulated cyclooxygenase (COX) enzyme activity as a major contributor to the pain experienced by women with primary dysmenorrhea. This is because COX aids in the production of prostaglandins (11).

The clinical feature of dysmenorrhea due to intense vasoconstriction includes cramping that typically begins a few hours before the onset of bleeding and can last for several hours or days after the onset of bleeding (12). The pain originates in the lower abdomen and may spread to the lower back and thighs (13). The pain is occasionally attributed to stretching of the peritoneum surrounding the ovary which also involves contraction-induced nerve impulses (14). Changes in bowel habits, nausea, exhaustion, dizziness, and headaches may be concomitant effects of dysmenorrhea (15).

In addition, vasopressin also contributes to vasoconstriction. Increased intrauterine pressure, vascular constriction, and reduced uterine blood flow, all contribute to tissue ischemia, leading to dysmenorrhea (16). The small afferent fibers originating from pelvic organs accompanied by sympathetic nerves are the carriers of pain information (17). However, any therapeutic strategy to suppress pain perception from these areas is challenging since the exact pathways of dysmenorrhea is unknown (18).

## 3. Approaches for treating dysmenorrhea

Several randomized, placebo-controlled studies have demonstrated that patients with presumed primary dysmenorrhea typically start their treatment with nonsteroidal anti- inflammatory medications (NSAIDs), which function as COX (cyclooxygenase) inhibitors (13). This in turn reduces the levels of prostaglandins and ceases tissue ischemia in the pelvic region (19). Transvaginal rings and patches reduce dysmenorrhea hence act as effective treatments for primary dysmenorrhea (20). Depot medroxyprogesterone acetate (an injectable progestin-only contraceptive) and oral contraceptives have also shown to be efficient for the treatment (21). The effectiveness of lifestyle-modification therapies in the management of dysmenorrhea has not been extensively studied. One crossover study comparing a low-fat vegetarian diet vs. placebo pill revealed that women in the intervention group experienced dysmenorrhea for shorter periods of time and with less intensity (22). Another double blind RCT studied thiamine, an amino acid elevating Vitamin B1, as a treatment among 500 women with moderate to severe symptoms of dysmenorrhea. The study showed significant relief of pain among the women in the intervention group compared to women in the placebo group (23). Another study successfully demonstrated the benefits of vasopressin-receptor as an antagonist for the treatment of dysmenorrhea (24). Psychotherapy, hypnotherapy, heat patches, and transcutaneous electrical stimulation are a few non-pharmacologic pain management techniques that are seen to be helpful (25). Surgical alternatives for pain relief include uterosacral ligament section and presacral neurectomy, however do not show a long-term pain alleviation (26). Further, surgical interventions can give rise to problems such as severe bleeding and constipation. These might be connected to the regrowth of nerves or the transmission of pain signals *via* other pathways (27). Therefore, surgical therapies for dysmenorrhea are typically not prescribed.

Hormonal contraception, topical heat, thiamine, vitamin E, or fish oil supplements; a low- fat vegetarian diet and acupressure are few viable alternatives that have been explored in several combinations (28).

## 4. Yoga as a complementary therapy

*Refer to Table 1 for further details*

**Table 1:** Review of literature on benefits of Yoga on Dysmenorrhea.

S.No.	Author- Year	Study Design	Sample Size	Intervention	Assessment Measures	Result
*Quasi-experimental Non-RCT*						
1.	Satyanand *et al*. 2014 (30)	Quasi- experimental nonrandomized controlled trial	Group A with dysmenorrhea + yoga (*n* = 50) Group B; control arm (*n* = 50	Experiment group: 45 min yoga for 3 months Control group: medication	VAS	VAS score decreased from 40.16 to 0.26 in the experiment group
2.	Nag *et al*. 2013 (31)	Quasi- experimental nonrandomized controlled trial	Students with dysmenorrhea and yoga (*n* = 60) Students in control group (*n* = 53)	Experiment group: 60 min yoga for 3 months Control group: PSS questionnaire	NPRS, PSS questionnaire of menstrual characteristic	88% of the experiment group showed significant (*p* < 0.0001) reduction in pain compared with no change in pain maong the control group.
3.	BR G *et al*. 2015 (29)	Quasi- experimental nonrandomized controlled trial	Students with dysmenorrhea- Group A (*n* = 45) Group B (*n* = 45) No control group	Experiment group A: Nadi shodhan Experiment group B: Kapal Bhati	ACPA-QOLS, MMDQ, NPRS	Significant (*p* < 0.001) improvement in quality of life and reduced prevalence of dysmenorrhea among both the groups after intervention practice
*Quasi-experimental Pre & Post*						
4.	Chien *et al*. 2013 (32)	Quasi-experimental before-and-after	Students with dysmenorrhea + yoga intervention group (*n* = 30) Control group (*n* = 30)	Experiment group: 30 min yoga for 8 weeks Control group: no intervention	SF-MDQ, level of homocysteine	Homocysteine levels decreased by 51.37% in the experiment group and 46.47% in the control group. After yoga intervention, overall MDQ scores considerably dropped (*p* < 0.05) in the experiment group.
5.	Parkhad *et al*. 2013 (33)	Quasi-experimental before-and-after	Students with dysmenorrhea & menstrual irregularities + yoga (*n* = 200)	Experiment group: 45 min yoga for 6 months Control group: no intervention	Investigator designed questionnaire of menstrual characteristics	Significant reduction in dysmenorrhea from 69.5% to 6.5% with *p* = 0.0032 among the study population.
*Randomised Control Trials*						
6.	Rakhshaee *et al*. 2011 (34)	RCT	Students with dysmenorrhea + yoga (*n* = 50) Control group (n = 42)	Experiment group: yoga at luteal phase Control group: no intervention	Menstrual characteristic questionnaire (eg, pain intensity and duration), VAS	Greater reduction in pain intensity (*p* < 0.05) in the intervention group as compared with the control group.
7.	Monika et al. 2012 (35)	RCT	Gynecologic patients with dysmenorrhea + yoga (*n* = 75) Control group (*n* = 75)	Experiment group: 35-40 min yoga for 6 months + conventional medication Control group: no intervention	Assessment of menstrual symptoms and pain through clinical history after 6 months	Significant (*p* < 0.05) reduction in dysmenorrhea
8.	Rani *et al*. 2011 (36)	RCT	Women with dysmenorrhea + yoga (*n* = 75) Control group (*n* = 75)	Experiment group: yoga nidra Control group: medication	SCAN	Significant reduction in pain (*p* < 0.006), gastrointestinal, cardiovascular and urogenital symptoms in intervention group as compared to control group.
9.	Sakuma *et al*. 2012 (37)	RCT	Women with regular menstrual cycle/dysmenorrhea + yoga (*n* = 67) Control group (*n* = 31)	Experiment group: yoga Control group: no intervention	VAS, GHQ-30	Participants reported reduced dysmenorrhea (*p* = 0.044) at 4 weeks. Decrease in GHQ score was observed that showed improvement in menstrual distress.
10.	Yang *et al*. 2016 (38)	RCT	Students with dysmenorrhea + yoga (n = 20) Control group (*n* = 20)	Experiment group: 60 min yoga for 12 weeks Control group: no intervention	VAS descriptive data for pain duration, SF-MDQ	Significant reduction in dysmenorrhea intensity (*p* < 0.001) and distress in intervention group and compared to the control group.

Yoga is being explored as a non-pharmacological, cost effective and feasible alternative that can benefit women with dysmenorrhea. It concentrates on aligning the body through gentle, focused movements along with improved breathing practices (39).

**Figure 2:**
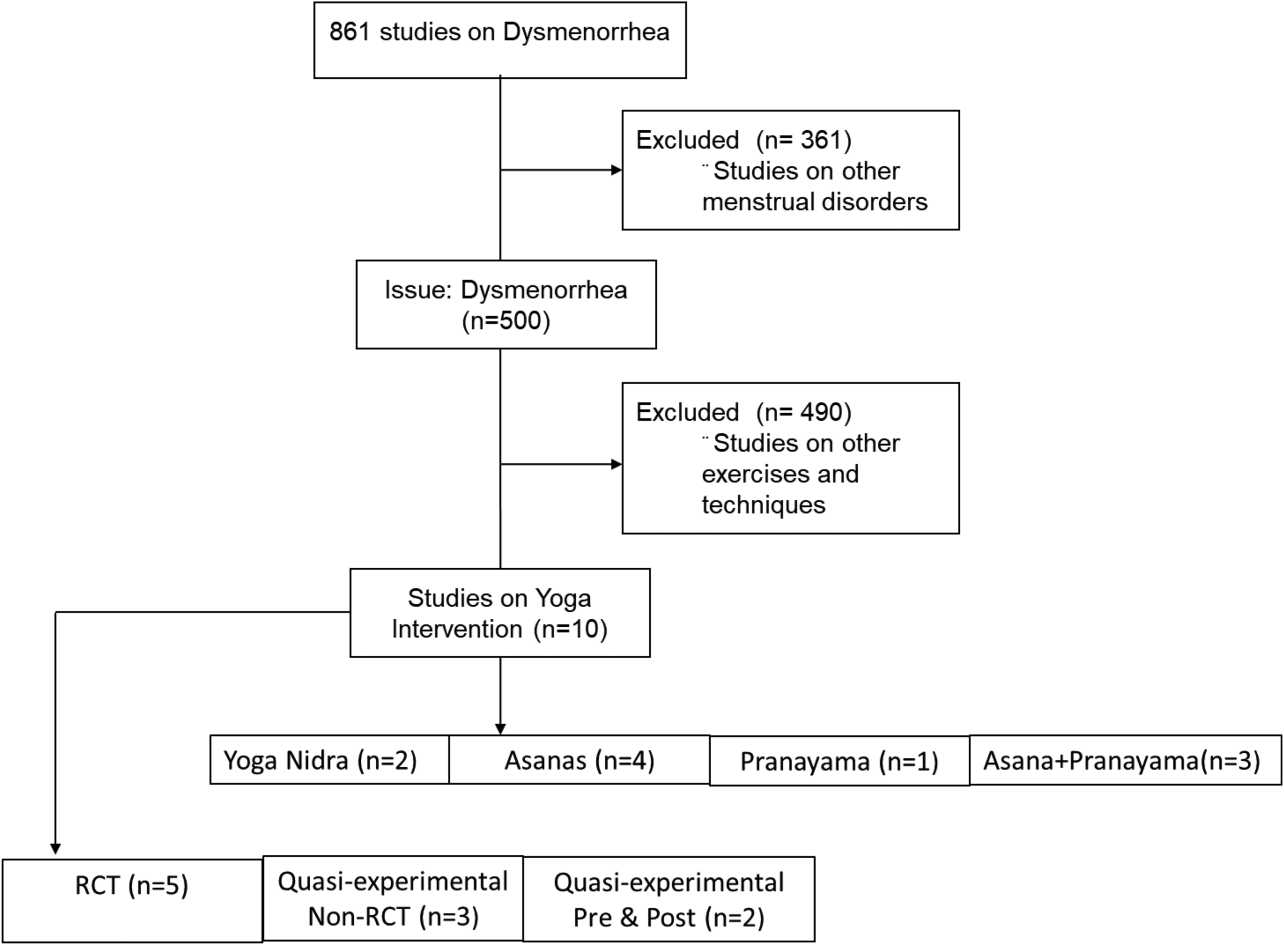
Flow diagram of research studies on dysmenorrhea.

A search was conducted to identify all studies that were undertaken from July 1990 to September 2022, to assess the effects of yogic practice on dysmenorrhea. These trials were published in various databases such as PubMed, Google Scholar and Scopus. These trials were published in various databases such as PubMed, Google Scholar and Scopus. The keywords selected were “yoga,” “menstrual pain,” or “dysmenorrhea.” The title search yielded a total of 861 studies. The review selection criteria were studies that included “yoga” as the intervention and “dysmenorrhea” as the issue. Studies on any other menstrual disorders apart from dysmenorrhea or if Yoga was not the intervention provided were excluded. Of the 861 studies only 10 studies met the selection criteria that involved participants with primary dysmenorrhea during the menstrual cycles in the past three months. The studies which included yogic intervention have been added in this review. The review search included all the study design to ensure a comprehensive literature on benefits of Yoga on dysmenorrhea (Figure 2).

The review included 1 study on *pranayama*, 4 studies on *asanas*, 3 studies on *asanas* and pranayama together; and 2 studies on *Yoga Nidra*.

### 4.1. Asanas on dysmenorrhea

*Refer to Table 1 for further details*

Sakuma et al*.* conducted a trial comprising 83 females in the yoga intervention group and 40 females in the control group. The reported change in pain at 2 weeks and at 4 weeks (after the intervention) served as the primary outcome while the 30-item General Health Questionnaire (GHQ30) score served as the secondary outcome measure. The results showed significant improvement in dysmenorrhea (*p* = 0.044) at 4 weeks along with reduced lower back pain during the intervention (*p* = 0.006). Decrease in GHQ score was observed that showed improvement in menstrual distress (37). A study by Rakhshaee et al. included 50 adolescent girls with primary dysmenorrhea who were randomized into a yogic intervention and 42 girls in a control module to study the effect of yoga on pain intensity, physical fitness and quality of life. The intervention included three yoga poses (Cobra, Cat and Fish). Visual Analog Scale (VAS) and Mann-Whitney test was used to assess the intensity for pain. The results of the study showed a significantly lower intensity of pain (*p* < 0.05) and pain duration in the experimental group from first to second month of intervention as compared to the control group. They also showed improved physical fitness and general health (34). Yang et al*.* conducted a clinical investigation on undergraduate girls with dysmenorrhea and studied the effect of *yogic asanas* on dysmenorrhea. 40 undergrad students selected at random were engaged in 12 h of practice, one hour every week. The intervention included *Surya namaskar, Bhujangasana, Marjariasana* and *Matsyasana* combined with relaxation and meditation. The results showed significant reduction in dysmenorrhea intensity (*p* < 0.001) and distress in the intervention group compared to the control group (38).

Another study was explored by Chien et al*.* to assess the effect of yoga intervention on a group of 35 women with primary dysmenorrhea compared to a group of 35 women as control. The women in the yoga group were provided with yoga intervention twice a week for 30 min, consecutively for 8 weeks. The cohort was administered a set of specific yoga asanas like *Ardha Kurmasana, Ardha Purvottanasana, Setu Bandha, Sarvangasana* and *Baddha Konasana*. The outcomes of the study were monitored by the level of homocysteine, an oxidative stress marker known to induce dysmenorrhea, and Menstrual Distress Questionnaires (MDQs). The results showed decrease in homocysteine level by 51.37% in experiment group and 46.47% in control group. After yoga intervention, overall MDQ score considerably dropped (*p* < 0.05) in the experiment group. The results suggested the therapeutic effect of yoga for dysmenorrhea (32).

Parkhad et al*.* conducted a study on 200 adolescent girls who experienced dysmenorrhea. They were given a yoga protocol of 45 min every day for 6 months which included *Swastikasana, Virasana, Padmasana, Gomukhasana, Paschimottanasana, Baddha konasana, Janu sirsasana, Trikonasana, Ardha chandrasana, Setu bandhasana, Supta Vajrasana* and *Savasana*. At the time of assessment after 6 months of practice, it was observed that there was significant reduction in dysmenorrhea from 69.5% to 6.5% with *p* = 0.0032 among the study population (33).

In a randomized controlled trial conducted by Satyanand et al., 100 female subjects with primary dysmenorrhea were studied. The study included 50 participants in the intervention group while 50 others in the control group. Females in the intervention group practiced *Sukshma vyayama, Padmasana, Paschimottanasana, Vajrasana, Ushtrasana, Shashankasana, Matsyasana, Uttanpadasana, Sarvangasana, Surya namaskara* followed by *Shavasana*. Both groups were evaluated for pain intensity using Visual Analog Scale (VAS) after three months. The result showed a decrease in VAS score from 4.16 to 0.26 in the experiment group indicating pain reduction post yoga intervention. The authors also suggested that yoga promotes physical relaxation by activating parasympathetic activity (30). Nag et al*.* studied the benefits of yoga on 113 girls who complained of primary dysmenorrhea and stress. The intervention cohort of 60 practiced *Navasana, Matsyasana, Dhanurasana, Vajrasana, Paschimottanasana, Ustrasana, Ardha matsyendrasana, Salabhasana, Bhujangasana, Sarvangasana, Uttanapadasana, Padmasana*, and *Surya namaskar* every day for 3 months under the supervision of a yoga trainer. 3 months later 88% of the experiment group showed significant (*p* < 0.0001) reduction in pain compared with no change in pain among the control group who did not receive any intervention (31).

**Figure 3:**
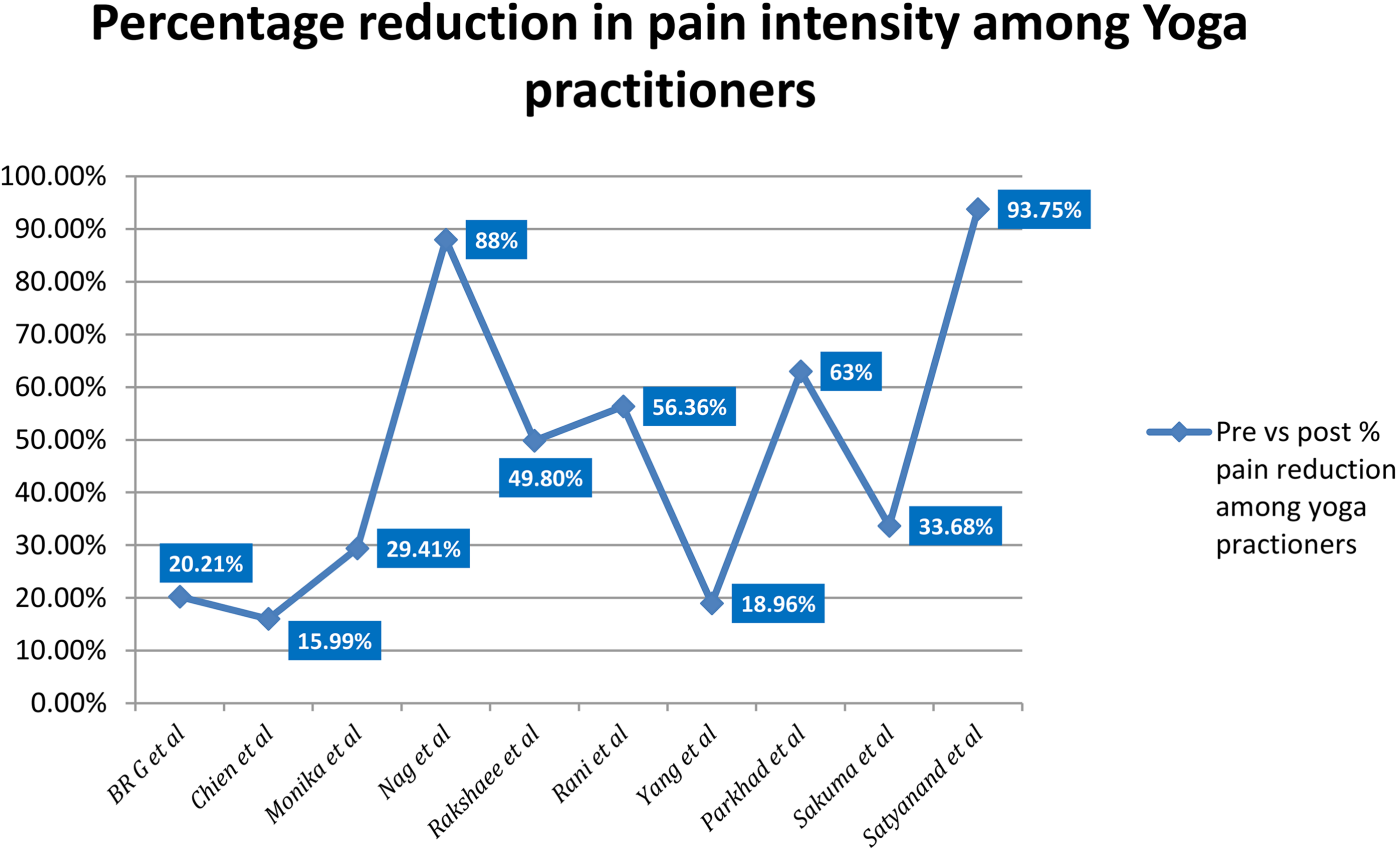
Graphical representation of percentage reduction in pain intensity among the yoga group.

The reviewed studies suggest that yoga can help reduce dysmenorrhea and related symptoms like anxiety and stress (Figure 3). Several yoga asanas and treatments, such as physical postures, breathing exercises, and relaxation methods, were regularly practiced over the course of many weeks or months that proved as effective complementary therapy for managing dysmenorrhea. The outcomes demonstrated the value of yoga intervention as a conventional therapy.

### 4.2. Yoga asanas and pranayamas on dysmenorrhea

*Refer to Table 1 for further details*

While asanas include one limb of Yoga, pranayama or breathing exercise are the other limbs of Yoga. Studies show deep breathing exercises (DBE) like *Kapalbhati, Bhastrika* and *Nadi Shodhan* pranayama are a set of breathing modules that can also act as an alternative form of treatment for primary dysmenorrhea (40). In a randomized control trial conducted by BR et al., 90 young women with primary dysmenorrhea between the ages of 18 and 25 were chosen at random to participate. The study group underwent yogic intervention consisting of *Nadi shodhan* in Group A and *Kapalbhati* in Group B for 30 days. The subjects were assessed at baseline and after 30 days for pain and distress using the Menstrual Distress Questionnaire (MMDQ), the American Chronic Pain Association's Quality of Life Scale and the numerical pain rating scale for pain. The results demonstrated the primary prevalence of dysmenorrhea among 65% of the population before the start of yogic intervention while the prevalence of dysmenorrhea significantly reduced post intervention (*p* value = <.001) with improvement in quality of life and reduced pain among the intervention group compared to the control group. The result suggested a decrease in the pain intensity with a parasympathetic activity following pranayama practice (29).

Pranayama, or breathing exercises are crucial component of yoga which can be used as a complementary therapy for various menstrual conditions. Studies show that certain breathing exercises can reduce pain and distress, and improve quality of life. Regular practice of pranayama can activate the parasympathetic nervous system, leading to reduced pain intensity and better overall well-being.

### 4.3. Yoga nidra on dysmenorrhea

*Refer to Table 1 for further details*

Rani et al*.* studied the effect of *yoga nidra* on 150 female subjects with menstrual irregularities and pain. The subjects were provided with 25 min of *yoga* nidra intervention for 6 months and assessment for pain was done using the Schedule for Clinical Assessment in Neuropsychiatry (SCAN) rating scale. The results showed a significant reduction in pain (*p* < 0.006), gastrointestinal, cardiovascular, and urogenital symptoms in the intervention group compared to the control group. The authors suggested the stimulation of the pituitary gland following the practice of *yoga nidra* produces compounds with analgesic properties, could be a probable mechanism of action for pain relief (36).

Monika et al*.* conducted a randomized control trial among 150 women with dysmenorrhea. The study measured hormone levels, as a marker of pain, in the subjects before and after 6 months of Yoga *Nidra* practice. Reduction in dysmenorrhea (*p* < 0.05) was observed in the intervention group (35).

In summary, practicing *Yoga Nidra* may reduce dysmenorrhea and related symptoms in women, possibly by stimulating the pituitary gland. These findings suggest that *Yoga Nidra* could be a useful complementary therapy for managing menstrual irregularities and pain in women. Further research is needed to investigate its long-term effects.

## 5. Mechanism of action for dysmenorrhea relief

As mentioned above, increase of vasoactive prostanoids leads to myometrial hyperactivity, reduced uterine blood flow and tissue ischemia all causing pain or dysmenorrhea during the cycle. Yoga and pelvic *asanas* are known to improve blood flow in the pelvic region thereby managing pain in the body (41). Further yoga also stimulates the release of beta- endorphins which are analgesics in nature (42). Studies have also shown the release of hormones like cortisol, glucose, plasma's renin, epinephrine and norepinephrine in the bloodstream as an effect of yoga that helps in regulating healthy body functions (43). Voluntary breathing control technique has been employed in many yogic interventions to decrease autonomic reactivity (44). Yoga also decreases sympathetic activity *via* the hypothalamic-pituitary-adrenal axis which plays an important role in contributing to pain reduction. Deep breathing and long exhalation relaxes the skeletal muscles in the body, especially the pelvic area and also contribute to pain reduction (45). A study suggests regulation of alpha brain waves, responsible for relaxation, pain relief and release of serotonin, favorably during yoga practice (46). One longitudinal study indicated that being overweight was a significant risk factor for both the likelihood of feeling dysmenorrhea and the increased duration of suffering (47). In two cross-sectional investigations, heavy or irregular periods were linked to a higher prevalence of dysmenorrhea (48). Four studies that investigated the association between smoking cigarettes and dysmenorrhea found that smokers had a higher risk of developing dysmenorrhea (49). According to Akerlund et al., cigarette smoking is known to cause vasoconstriction. He demonstrated reduced endometrial blood flow in women with dysmenorrhea using a thermistor probe technique (50). Many studies have also found a correlation between perceived stress linked to work or general life events and the prevalence of dysmenorrhea among the psychological factors investigated.

Improved body circulation with good physical and mental health has been highlighted as key factors in reducing dysmenorrhea and experiencing a healthy menstrual cycle.

## 6. Conclusion

Menstruation is an integral part of every woman's life cycle influencing the physical, mental, emotional health and quality of life. Unfortunately, women's health is not much attended in society. Many menstrual disorders including dysmenorrhea go unrecognized and untreated. With women's health gaining increased attention, several cost-effective, alternate salubrious interventions are being investigated to treat the women's health disorder. A significant amount of literature supports the benefits of Yoga (*asanas/pranayama*/*Yoga Nidra*) in reducing the dysmenorrhea. While few studies have demonstrated the positive effects of Yoga in treating dysmenorrhea and several more on other menstrual disorders, more studies with good scientific rigor can promote yoga as evidence based solution in treating menstrual health problems.

## Author contributions

All authors contributed to the article and approved the submitted version.

## Conflict of interest

The authors declare that the research was conducted in the absence of any commercial or financial relationships that could be construed as a potential conflict of interest.
